# Economic, environmental and social indicators of sustainability among smallholders in Ethiopia: Based on tool for agroecological performance evaluation data

**DOI:** 10.1016/j.dib.2023.109988

**Published:** 2023-12-18

**Authors:** Muluken G. Wordofa, Chanyalew S. Aweke, Getachew S. Endris, Getachew N. Tolesa, Tesfaye Lemma, Jemal Y. Hassen, Dario Lucantoni, Anne Mottet

**Affiliations:** a School of Rural Development and Agricultural Innovation, Haramaya University, Ethiopia; b Haramaya Institute of Technology, Haramaya University, Ethiopia; c Animal Production and Health Division (NSAG), Food and Agriculture Organization of the United Nations (FAO), Rome, Italy; d International Fund for Agricultural Development (IFAD), Rome, Italy

**Keywords:** Agroecology, Performance, Sustainable development, Smallholder farmers, Ethiopia

## Abstract

This data article is a result of research conducted by a multidisciplinary team of researchers with the aim of analyzing agroecological transition and performance of agroecology in Ethiopia. It was conducted in four districts of Oromia and Southern Nations, Nationalities and People's (SNNP) regional states - Fedis district (East Hararghe Zone) and Miesso district (West Hararghe Zone) from the Oromia region, and Kindo Koysha district (Wolaita Zone) and Meskan district (Gurage Zone) of SNNP region. The rationale behind generating this dataset lies on the fact that there is scanty empirical evidence on the multidimensional performance of agroecology in the country. Available evidence only provides data on limited indicators of sustainability. Hence, there is a lack of comprehensive data on the economic, environmental and social indicators of sustainability and agroecological transition in the context of smallholder farming systems in the country. To fill this gap, the Food and Agriculture Organization of the United Nations (FAO) commissioned a consultancy project that employed the Tool for Agroecological Performance Evaluation (TAPE) to assess several dimensions and indicators of agroecological transitions and generate globally comparable data. A random sample of 619 farms were selected from 12 Kebeles (i.e., the lowest administrative unit), and trained enumerators gathered primary data based on a modified TAPE questionnaire using Kobo Toolbox. Participation of smallholders was on a voluntary basis and informed consent was obtained from the respondents. The survey questionnaire contained information on basic socio-economic and demographic characteristics, access to services and infrastructure, livelihood and Income-Generating Activities (IGAs), social and ecological indicators. Data on the 10 elements of agroecology was also collected. The collected data were entered into a STATA software, cleaned and analyzed through descriptive and inferential statistics. The outputs were summarized in Tables, Charts and Graphs. Since the data contained in this data article are disaggregated by study district, categories of agroecological transition, production typology and land size groups, this can foster the promotion of specific projects and programs that can address expressed needs of smallholder farmers. It can also facilitate agro-ecological based implementation of development interventions to encourage agroecological transition, sustainable development and food systems. The dataset can also enable researchers, practitioners and other decision-makers to make comparative analysis on the economic, environmental and social dimensions of sustainability. The analyzed data is provided in this data article. The raw data used to prepare figures is provided as a supplementary material. A copy of the questionnaire, raw dataset, and description of variables are available online on Mendeley Data.

Specifications Table

**Table:** 

Subject	Agricultural Sciences
Specific subject area	Agroecology and food systems providing data on economic, environmental, and social indicators of sustainability in primarily mixed and agropastoral farming systems.
Data format	Raw, Analyzed
Type of data	Raw data: .csv file (data on 178 variables for 619 farms) .pdf file (codebook containing description of variables) Analyzed data: Table, Chart/Graph, Figure
Data collection	This dataset contains economic, environmental, and social indicators of sustainability. The data were gathered using the Tool for Agroecological Performance Evaluation (TAPE) developed by the FAO [1]. Primary data on basic socio-demographic, economic, institutional, social and ecological dimensions of agroecological performance were generated using a survey questionnaire uploaded on Kobo Toolbox (on a smart device). The data collection and overall field work were supported by the FAO, and supervised by the researchers and local research translation partners. The data contained herein refers to a randomly selected 619 smallholder farms located in four districts of Ethiopia.
Data source location	Haramaya University (eastern Ethiopia) and FAO are the owners of the data described herein. The field-based data collection was carried out in Oromia and Southern Nations, Nationalities and People's (SNNP) regions of the country.
Data accessibility	Repository name: Mendeley Data Data identification number: DOI: 10.17632/tsdp553dsm.2 Direct URL to data: https://data.mendeley.com/datasets/tsdp553dsm/2

## 1 Value of the Data

• The data presented here can be used by food system actors and researchers interested in understanding the contribution of agroecology to transforming current agriculture and food systems.
• The data provides an important description of the Characterization of Agroecological Transition (CAET) at farm, household, and community levels based on the 10 elements of agroecology.
• These datasets can be reused by researchers, graduate students, local-level practitioners, NGOs, and decision-makers to assess agroecological transition levels of farm households and production systems; examine strengths and weaknesses of the production systems and farms; analyze how the elements of agroecology interact and their relative importance for the overall process of agroecological transition; and identify pathways to support the transition process.
• The data can also be used to demonstrate the transformative capacity of agroecology and its broad applicability to food system change. More specifically, it can be used to inform priority setting, targeting and resource allocation decisions.

## 2 Background

The compilation of the dataset was undertaken as part of an agroecology survey with the Food and Agriculture Organization (FAO) to assess agroecological transition levels of farms in Ethiopia using the Tool for Agroecology Performance Evaluation (TAPE). The implementation of the TAPE tool is the first of its kind in Ethiopia. The preparation of the survey questionnaire (the TAPE tool) draws the theory and practice of agroecology – conceptualized as the integrative study of the ecology of the entire food system, encompassing ecological, economic, and social dimensions seeking to contribute to transforming food systems by ensuring a regenerative use of natural resources and ecosystem services [2].

Data presented in this data article came out of a carefully planned and rigorous field survey across two regional states in Ethiopia. The motivation behind the compilation of this dataset in its current format is aimed at provide researchers, students, local-level practitioners, NGOs, and decision-makers at various levels with rich data to analyze levels of agroecological transition of farms and production systems at farm, households and community levels. The result can be used as an entry point to help design project interventions to support the transition of smallholder producers to agroecology.

## 3 Data Description

**Fig. 1:**
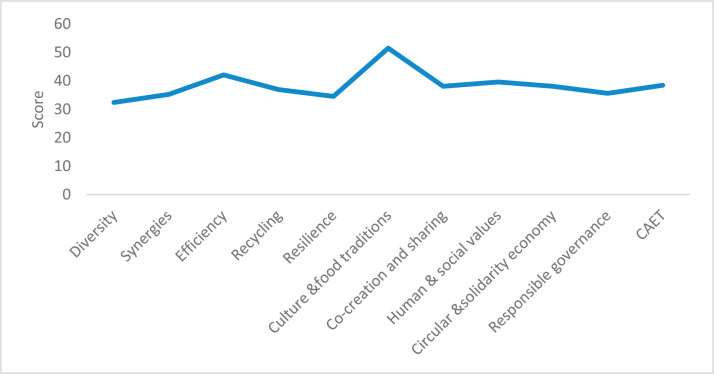
CAET score for the full sample and individual scores for the 10 elements characterizing agroecological transition (mean values).

**Fig. 2:**
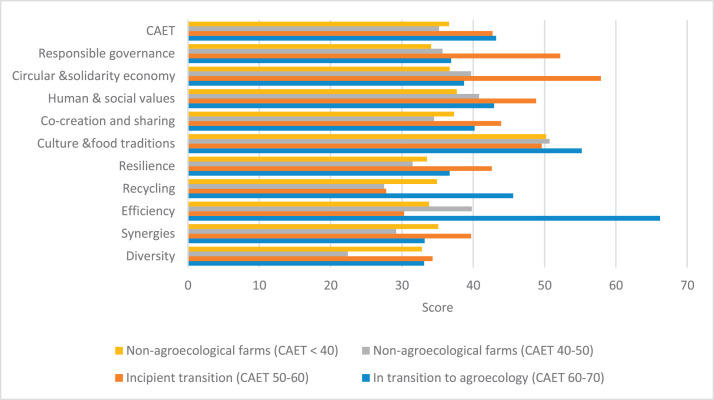
Categorization of the 619 farms into four categories of agroecological transition based on the CAET score (mean values).

**Fig. 3:**
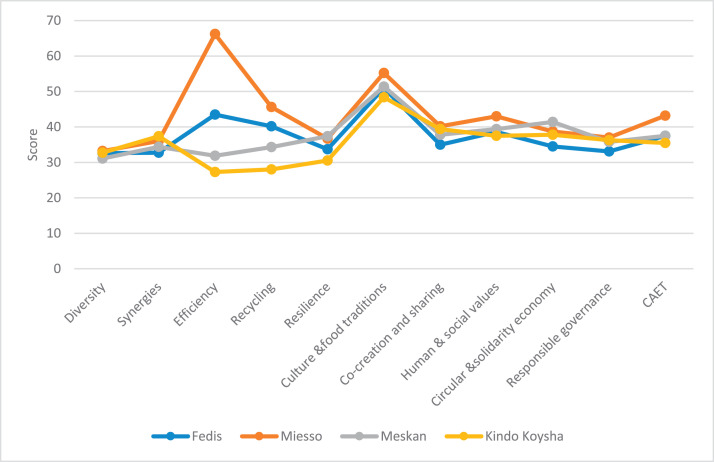
Distribution of CAET score across the four study districts (mean values).

**Fig. 4:**
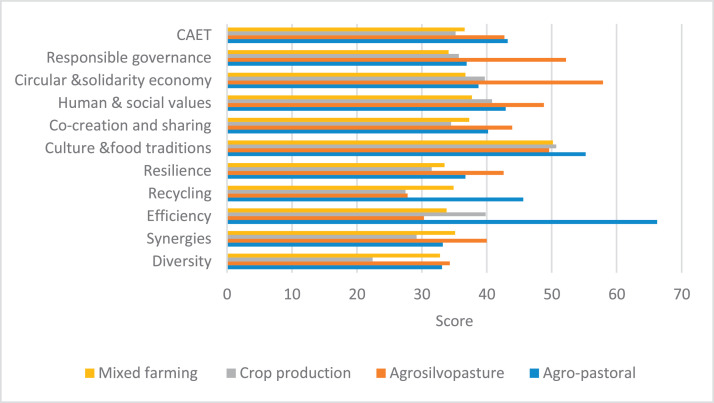
Distribution of CAET score among the four farm typologies/farming systems in the study area (mean values).

**Fig. 5:**
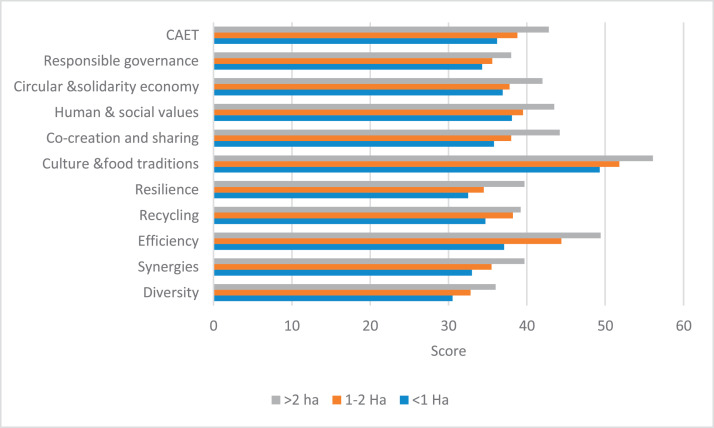
Distribution of CAET scores across different land size categories (mean values).

**Fig. 6:**
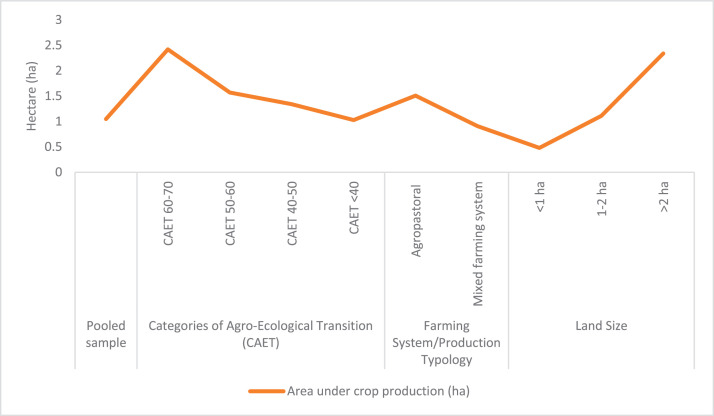
Area under crop production (ha) disaggregated by CAET, farm typology and land size groups.

**Fig. 7:**
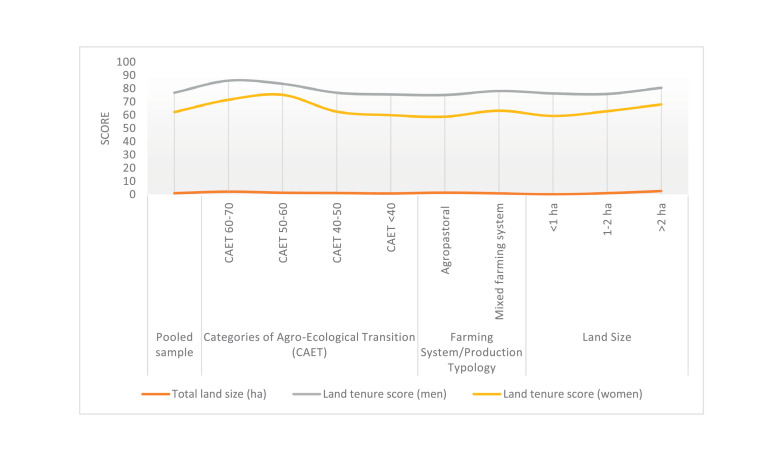
Average land size (ha) and land tenure score for male-headed and female-headed households (mean) disaggregated by study District, CAET, farming system, and land size categories.

**Fig. 8:**
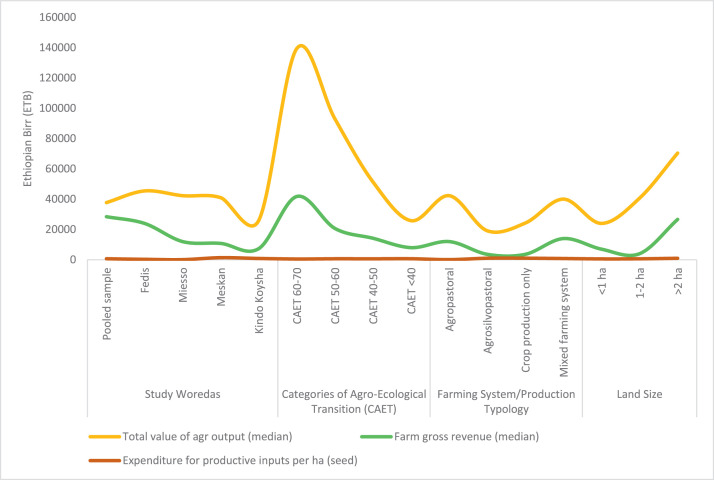
Total value of agricultural output, expenditure and farm gross revenue in birr by study District, CAET, typology of production, and land size categories.

**Fig. 9:**
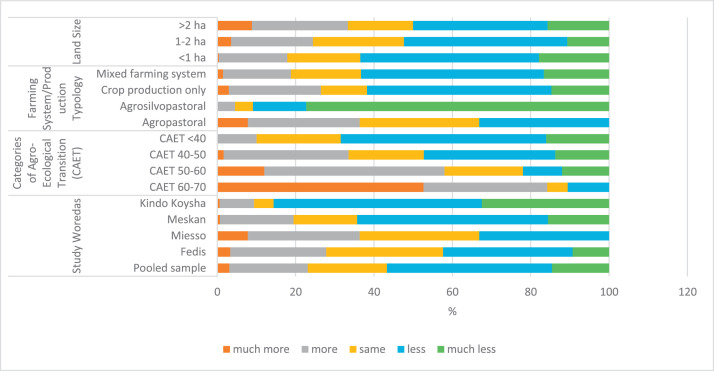
Perception of farm revenue by study District, CAET, production typology, and land size categories (%).

**Fig. 10:**
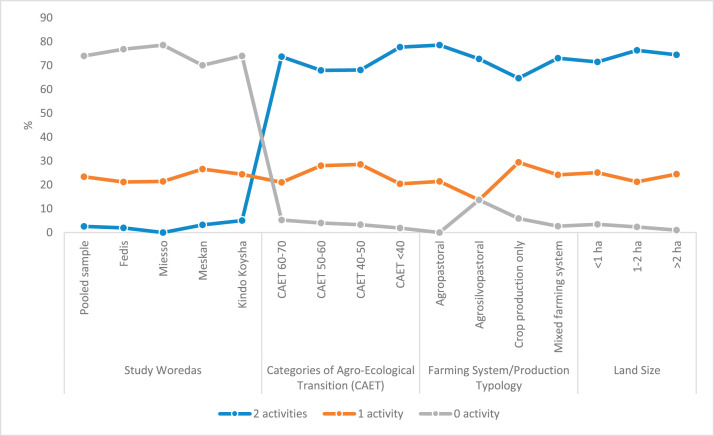
Engagement in other off-/non-farm income-generating activities (IGAs) by study District, CAET, farming system, and land size categories (%).

**Fig. 11:**
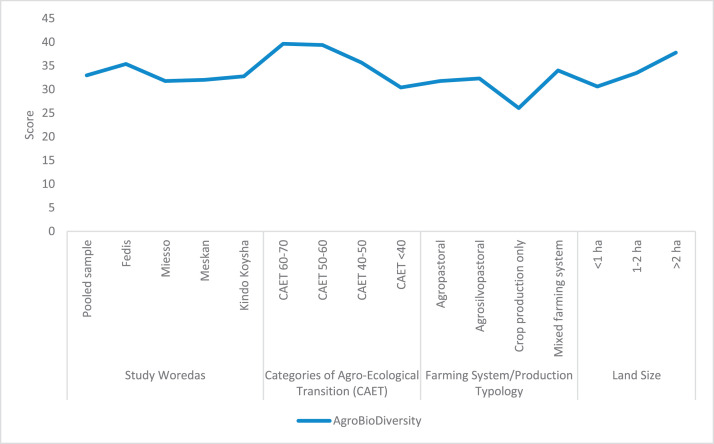
GINI-SIMPSON overall agrobiodiversity score by study District, CAET, farming system, and land size categories (mean values).

**Fig. 12:**
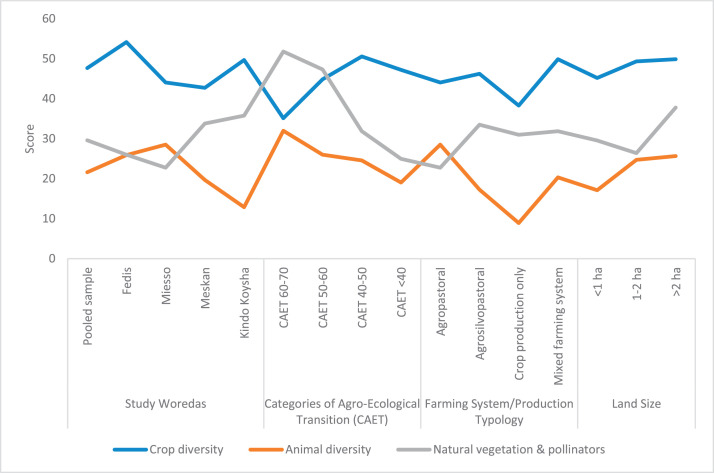
Components of agrobiodiversity (i.e., crop, animal, and natural vegetation and pollinators) disaggregated by study District, CAET, production typology, and land size categories (mean values).

**Fig. 13:**
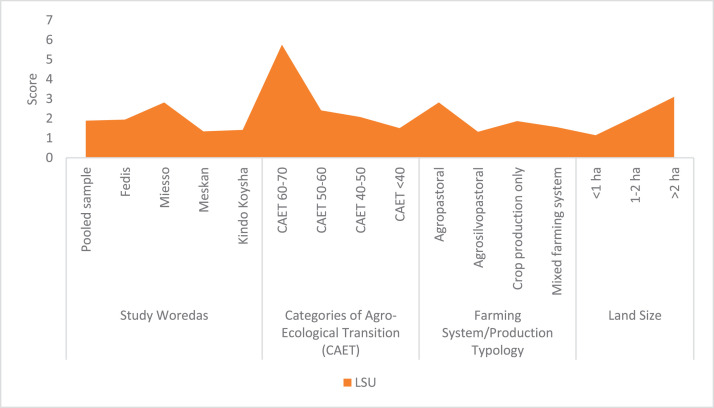
Livestock Standard Unit (LSU) disaggregated by study District, CAET, farming system, and land size categories (mean values).

**Fig. 14:**
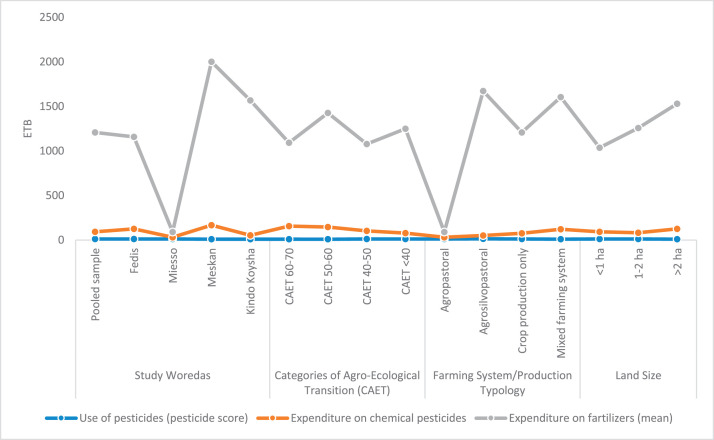
Use of chemicals in agricultural production activities disaggregated by study District, CAET, farm typology, and land size categories (mean values).

**Fig. 15:**
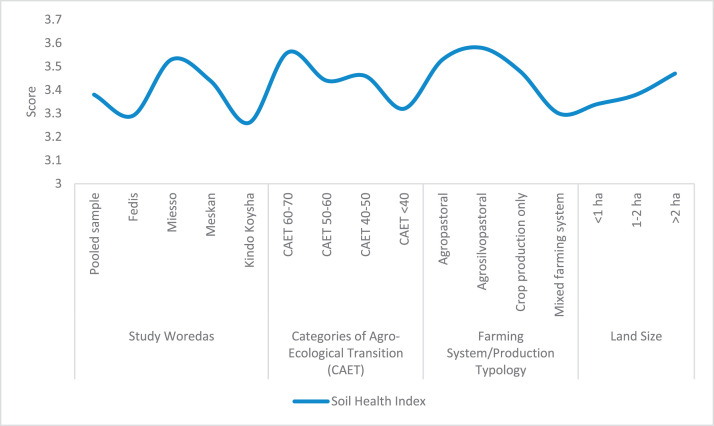
Soil health index by study District, CAET, farm typology, and land size categories (mean values).

**Fig. 16:**
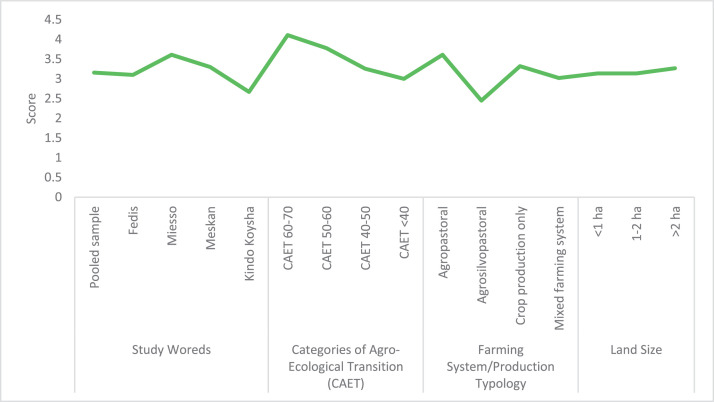
Minimum Dietary Diversity Score for Women (MDDSW) disaggregated by study area, CAET, typology of production system, and land size categories (mean values).

**Fig. 17:**
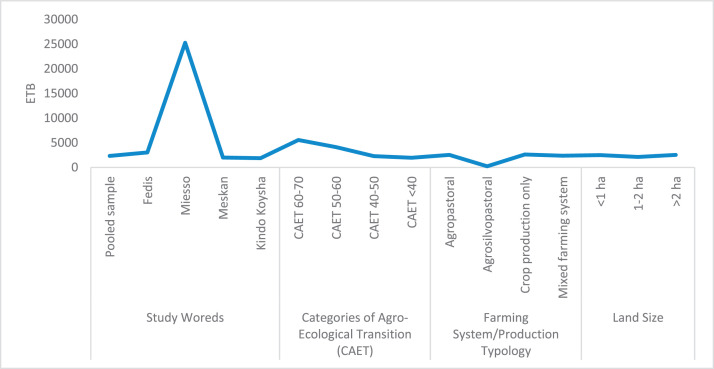
Expenditure for food per person (Ethiopian Birr (ETB), mean values).

**Fig. 18:**
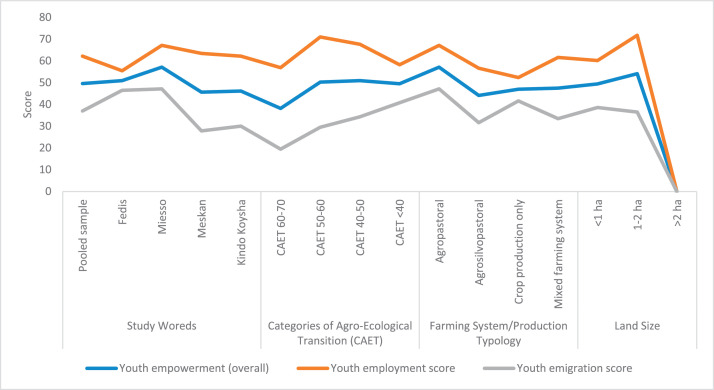
Youth empowerment score (employment, emigration and overall score) by study area, CAET, farming system, and land size categories.

**Fig. 19:**
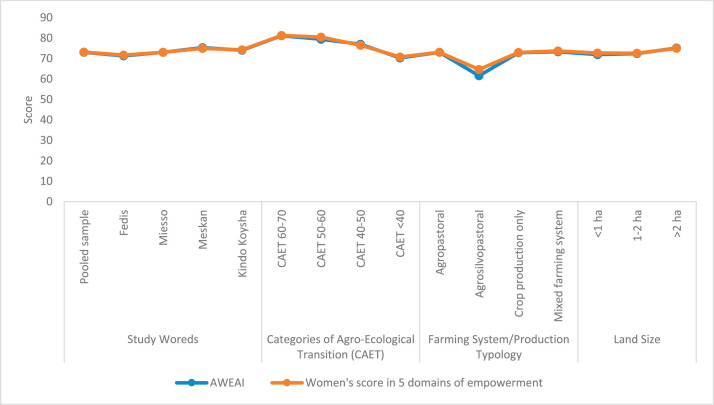
Abbreviated Women's Empowerment in Agriculture Index (AWEAI) and women's score in five domains of empowerment disaggregated by study District, CAET, farming system, and land size categories.

**Fig. 20:**
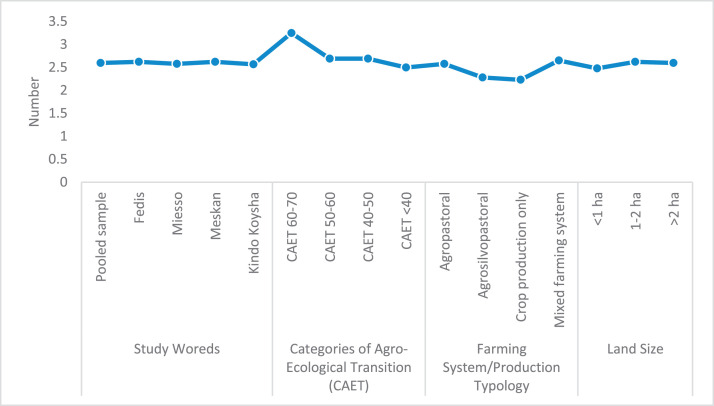
Average number of family labor involved in agricultural production disaggregated by study District, CAET, farming system, and land size categories.

The raw and analyzed data refer to various indicators of economic, environmental, and social dimensions of sustainability pertaining to smallholder farming systems in Ethiopia. The analyzed data in this data article were presented for the full sample (i.e., 619 farms) as well as disaggregated by district/District, Characterization of Agroecological Transition (CAET) categories, farm typology, and land size groups. Fig. 1 presents the average CAET score for the full sample and the individual scores for the 10 elements characterizing agroecological transition. The CAET score was used to categorize the studied farms into four categories (Fig. 2). Fig. 3 presents the distribution of the CAET score across the four study districts. Furthermore, the CAET scores were disaggregated by the prevailing farming systems (i.e., farm typology) as indicated in Fig. 4. Finally, the CAET scores disaggregated by land size categories were presented in Fig. 5. Economic indicators of sustainability, disaggregated by CAET, production typology and land size group, are presented in Fig. 6 (area under crop production), Fig. 7 (total land holding and tenure security), Fig. 8 (production, revenue and expenditure for inputs), Table 1 (household expenditure), Fig. 9 (perception of revenue), and Fig. 10 (involvement in off-/non-farm income-generating activities). Environmental indicators of sustainability are presented in Fig. 11 (agrobiodiversity index), Fig. 12 (crop, animal and natural vegetation diversity indices), Fig. 13 (Livestock Standard Unit), Fig. 14 (use of agrochemicals), and Fig. 15 (soil health index). Social dimensions of sustainability are presented in Fig. 16 (Minimum Dietary Diversity Score for Women – MDDSW), Fig. 17 (food expenditure per person), Fig. 18 (youth empowerment – employment and emigration scores), Fig. 19 (women's empowerment), and Fig. 20 (family labor employed in own farm). The survey questionnaire, codebook describing the variables in the dataset, and raw data are available online on Mendeley Data [3].

**Table 1:** Household expenditures for inputs; energy, machinery and maintenance and finance disaggregated by study district (Ethiopian Birr (ETB), median values, unless stated).

	Fedis	Miesso	Meskan	Kindo Koysha	Total
Expenditures for inputs					
Food for self-consumption	10,000	3,000	7,000	5,000	7,000
Seeds	302.25 ^a^	95.36 ^a^	1,000	500	250
Fertilizers	900	59.38 ^a^	1,660	1,000	850
Feed	973.18 ^a^	243.83 ^a^	731.17 ^a^	768.31 ^a^	678.56 ^a^
Veterinary services	50	120	60	277.5	120
Livestock purchases	2988.08 ^a^	1991.88 ^a^	2424.79 ^a^	1,151.22	2125.30 ^a^
**Energy, machinery and maintenance**					
Maintenance, rental of machineries	350.54 ^a^	42.45 ^a^	87.73 ^a^	96.61 ^a^	142.87 ^a^
Fuel	134.97 ^a^	462.34 ^a^	409.74 ^a^	230.69 ^a^	309.52 ^a^
Energy	428.58 ^a^	738.96 ^a^	566.43 ^a^	295.72 ^a^	505.75 ^a^
Transportation	200	207.26 ^a^	110	180	120
**Financial information**					
Taxes	350.54 ^a^	42.45	87.73 ^a^	96.61 ^a^	120
Subsidies received	134.97 ^a^	462.34 ^a^	409.74 ^a^	230.69 ^a^	226.53 ^a^
Interest on loans	428.58 ^a^	738.96 ^a^	566.43 ^a^	295.72 ^a^	296.45 ^a^
Cost of renting land	200	207.26 ^a^	110	180	372.94 ^a^

Source: Researchers’ Own Illustration (2022). ^a^ since the median values are zero, the mean values in these cases are kept.

Note: 1 US$ = 47.4603 Ethiopian Birr (ETB) on November 10, 2021.

### 3.1 Characterization of agroecological transition (CAET)

The average scores for CAET and 10 elements characterizing agroecological transition [4,5] are given in Fig. 1. The average CAET score for the 619 farms studied was found to be 38.4. Among the 10 elements, ‘*Culture and Food Traditions*’, ‘*Efficiency’*, and ‘*Human and Social Values*’ had the largest scores.

The studied farms were classified into four based on the cut-off points established for the CAET score (Fig. 2). This Fig. also provides the scores for the 10 elements of agroecology for the different types of farms.

In terms of CAET scores disaggregated by study district, Fig. 3 shows that Miesso had a relatively larger overall CAET score and Kindo Koysha had the lowest score.

The CAET scores and the scores for the 10 elements of agroecology for the different farming systems in the study area are given in Fig. 4. Whereas the largest CAET scores are associated with the agropastoral production typology, the lowest CAET scores are found in the crop production only system.

In terms of land size, the CAET scores indicate that there is an increasing trend with an increase in land holding size. A similar pattern of increment in CAET scores is observed for all the 10 elements of agroecology with an increase in land size (Fig. 5).

### 3.2 Economic indicators of agroecological performance

The economic indicators of agroecological performance in the study area considered elements, such as area under crop production; land tenure security; value of agricultural outputs, revenue and expenditure for productive inputs; household expenditure for other inputs; perception of revenue; and engagement in off-/non-farm Income-Generating Activities (IGAs). As indicated in Fig. 6, data on area under crop production, measured in hectare, is provided for the pooled sample (i.e., 619 farms) and then disaggregated by the categories of agroecological transition (CAET), dominant farming systems, and land size groups.

In terms of tenure security, Fig. 7 shows the land tenure scores for male-headed households and female-headed households. These scores are disaggregated by the study district to show variations among the study areas. Furthermore, the land tenure scores are provided for the categories of agroecological transition, farm typology, and land size groups.

The data on agricultural production, revenue and expenditure for productive inputs are provided in Fig. 8. The Fig. depicts the data for the pooled sample, and for each study district, CAET categories, farming systems, and land size groups.

Table 1 provides district level data on expenditure for agricultural inputs, energy, machinery and maintenance. It also provides financial information related to tax, subsidy, cost of renting land and interest on loans.

The data on household's perception of farm revenue, based on a five-point Likert Scale measurement, are given in Fig. 9. This data relates to household's perception of current income compared to their income five years ago. Farmers in Kindo Koysha and Meskan districts reported lesser income compared to those in the other districts. Farmers in more advanced agroecological farms reported better income compared to those in non-agroecological farms. The mixed and crop production only farming systems reported lesser income.

The level of involvement of smallholder farmers in off-/non-farm IGAs is presented in Fig. 10. The data shows that about 74% of the respondents did not participate in any IGA during the last cropping season. However, large proportion of farmers in non-agroecological farms and in agropastoral farming systems are found to participate in more than two IGAs.

### 3.3 Environmental indicators of agroecological performance

The environmental indicators of sustainability presented here include: agrobiodiversity index; indices of crop, animal and natural vegetation; Livestock Standard Unit (LSU); use of chemicals in agriculture; and soil health index.

In relation to agrobiodiversity index [6], Fig. 11 shows that the average GINI-SIMPSON agrobiodiversity index is found to be 32.97 (i.e., very low level of diversity of crops, animals, and natural vegetation and pollinators). The largest agrobiodiversity index is associated with farms located in Fedis district, farms in transition to agroecology (i.e., more advanced agroecological farms), mixed farming systems, and larger farms. The index decreases from more agroecological to non-agroecological farms. Crop production only system is also characterized by less agrobiodiversity index.

Disaggregating the overall agrobiodiversity index into its components, Fig. 12 depicts the indices for crop, animal, and natural vegetation and pollinators. In terms of crop diversity, the largest indices are found in Fedis district, non-agroecological farms, mixed farming systems, and larger farms. The largest animal diversity is found in Miesso district, more advanced agroecological farms, agropastoral farming systems, and larger farms. The largest indices for natural vegetation and pollinators are associated with Kindo Koysha district, more advanced agroecological farms, agrosilvopastoral farming systems, and larger farms.

The Livestock Standard Unit (LSU) data (Fig. 13) indicates that the largest values are found in Miesso district, farms more advanced in agroecological transition, agropastoral farming systems, and larger farms.

The data on use of chemicals in agricultural production (Fig. 14) shows pesticide score, expenditure on pesticides and fertilizers. For instance, the largest expenditure for fertilizer is found in Meskan district, farms in incipient transition to agroecology, agrosilvopastoral farming systems, and larger farms. Likewise, the largest expenditures on chemical pesticides are found in Meskan district

The data on soil health index [6], depicted in Fig. 15, indicates that the average soil health index for the 619 farms is 3.38. Furthermore, the data shows that Miesso district, more agroecological farms, agrosilvopastoral farming system, and larger farms have better soil health indices.

### 3.4 Social indicators of agroecological performance

The data on social indicators of sustainability refers to Minimum Dietary Diversity Score for Women (MDDSW), expenditure for food per person, youth empowerment (employment and emigration), women's empowerment, and family labor employed on one's own farm.

In relation to dietary diversity [7,8], the average MDDSW is found to be 3.16. The MDDSW is found to be higher for women in Miesso district, managing more advanced agroecological farms, making a living in agropastoral farming systems, and possessing larger land size (Fig. 16).

In relation to food consumption, the expenditure for food per person is given in Fig. 17. It indicates smallholder farmers’ food expenses (measured by Ethiopian Birr (ETB) by district, CAET category, farm typology and land size group.

The data on youth empowerment, employment and emigration scores [6] is given in Fig. 18. The largest youth empowerment and employment scores are found in Miesso district, farms in incipient transition to agroecology, agropastoral farming system, and among farmers with 1-2 ha of land. The data further shows that youth proneness to emigrate sharply decreases from non-agroecological to agroecological farms.

In relation to women's empowerment [9], the mean Abbreviated Women's Empowerment in Agriculture Index (AWEAI) is found to be 73.15. The data further shows that the largest AWEAI are associated with Meskan district, more advanced agroecological farms, agropastoral farming systems, and larger farms (Fig. 19). A similar pattern is also observed for women's score in five domains of empowerment.

The data on family labor employment in one's own farm (Fig. 20) indicates that more advanced agroecological farms employ more family members compared to non-agroecological farms. Furthermore, the data shows that mixed farming system and large land holding size offer more on-farm employment opportunities for family labor.

## 4 Experimental Design, Materials and Methods

### 4.1 Sampling and study design

**Fig. 21:**
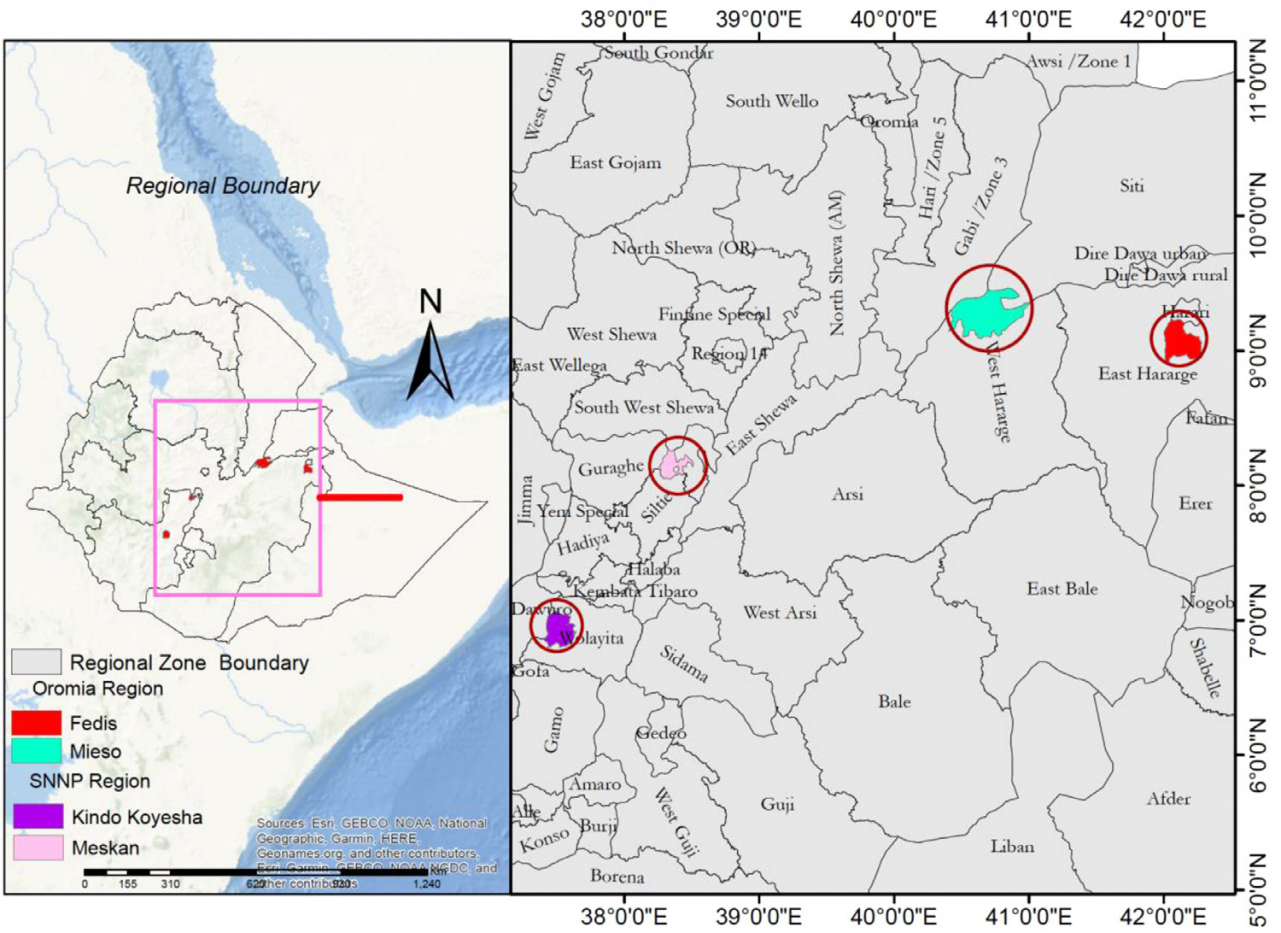
Map of the study area showing sampled districts in the two regions.

This study was conducted in four FAO pre-selected Districts of Oromia and SNNP regions. These are: Fedis District (East Hararghe Zone) and Miesso District (West Hararghe Zone) from the Oromia region, and Kindo Koysha District (Wolaita Zone) and Meskan District (Gurage Zone) of SNNP region (Fig. 21).

In each district, three representative Kebeles (i.e., the lowest level of administration in Ethiopia) were selected in consultation with the FAO technical team, regional and District administrators, and Development Agents (Table 2). A total of 619 sample respondents were selected (305 farm households from the Oromia region and 314 respondents from the SNNP region). A list of households was obtained from the District facilitators, and selection was done randomly.

**Table 2:** Kebeles selected from each district.

Region/District Kebele	Oromia region		SNNP region	
	Fedis	Miesso	Meskan	Kindo Koysha
1	Risqi	Oda Bella	Elle	Dada Kare
2	Tuta Kanisa	Gorbo	Bati Futo	Fachena
3	Muleta	Sodoma	Weja Bati	Sere Finchawa

### 4.2 Data and methods

There are a range of tools and techniques to assess agroecological performance and transition. A recent review by Darmaun et al. [10] provides a description for 14 assessment techniques. From these, the Tool for Agroecology Performance Evaluation (TAPE), developed by FAO, was employed to assess the multidimensional agroecological performance of farms and their transition levels. The detailed guideline describing the TAPE methodology is available in FAO [1] and in Mottet et al. [6]. The TAPE, combined with the Kobo Toolbox mobile/cell phone-based application, was employed using Tablets to generate the necessary information from households and individuals. The generic TAPE questionnaire has been contextualized to the Ethiopian situation in collaboration with the FAO team. For instance, ‘location’ variable was made to refer to ‘zone’, ‘district’ and ‘Kebele’; ‘age’ was contextualized to refer to the age structure in Ethiopia (e.g., 15-29 youth); some more options were added to ‘intended destination of agricultural production’; the variables under ‘culture and food traditions’ were made to refer to the study areas’ contexts; and the food types used to compute dietary diversity were made to refer to the local food production diversity. Despite these modifications, the original contents of the standard TAPE questionnaire were not significantly altered. This has enabled the research team to generate data on the 10 elements of agroecology following standard scientific approaches and practices. The finalized version of the questionnaire was formatted and uploaded to Kobo Toolbox by the FAO TAPE Team. The TAPE questionnaire was translated into local languages (Amharic and Afan Oromo). The TAPE questionnaire was pretested and validated at a randomly selected rural household. Each enumerator was supported by the technical team and the facilitators from the District and Kebele during the onset of the interviews. The technical team assisted the enumerators throughout the pre-testing process and received instant feedback and clarification on some items contained in the TAPE questionnaire. Data collected included socio-economic, environmental and demographic characteristics and contexts of the production systems, such as location, household size, household assets, agro-ecological zone, land use patterns, topography, forests, access to land, and agricultural commodities produced. More specifically, data were collected on the 10 elements of agroecology [4,5].

Both descriptive (percentages, mean, standard deviation) and inferential statistics were used to present summary of the data. The STATA software and other appropriate platforms (e.g., excel spreadsheet) were used for the analysis of quantitative data. Indices were developed as part of the analysis in collaboration with the FAO TAPE team. For instance, the Average Women's Empowerment was computed in Agriculture Index (AWEAI) was constructed and categorized as (Green (desirable): A-WEAI ≥80%; Yellow (acceptable): A-WEAI ≥60% and <80%; Red (unsustainable): A-WEAI <60%). The team also computed the overall CAET and indices for the 10 elements of agroecology (i.e., Diversity, Synergies, Efficiency, Recycling, Resilience, Culture and food Traditions, Co-Creation & Sharing, Human & Social Values, Circular and solidarity Economy, and Responsible Governance). Based on the CAET score, farms were classified into four: transition to agroecology (60-70), incipient transition (50-60), non-agroecological (40-50), and non-agroecological (<40). The Gini-Simpson Agrobiodiversity Index, Crop Diversity Index, Animal Diversity Index, and Others Diversity Index (i.e., natural vegetation, trees and pollinators) were also computed. The index was further categorized into desirable (≥70%), acceptable (≥50 & <70), and unsustainable (<50). Finally, a Soil Health Index was also computed. In addition to the above indices, various scores were calculated for items such as land tenure, youth employment and emigration, and women's score in five domains of empowerment. Minimum Dietary Diversity Score for Women (MDDSW) was computed following the guideline prepared by FAO and the USAID FANTA Project ([8]; FAO and [7]). For youth employment and emigration scores, a score of 0 has been assigned to situations considered individually unfavorable and 1 to those considered favorable. A score of 0.5 is given to intermediate situations. The following thresholds are used for the final average score of employment and emigration: Green (desirable): Score ≥70%; Yellow (acceptable): Score ≥50% and <70%; and Red (unsustainable): Score <50% [1]. Furthermore, analysis of performance by CAET categories, typology of farms, land size categories, and District was carried out to understand the influence of agroecological transition on the performance of farms and other indicators of sustainability. In some instances, analysis was disaggregated by gender (e.g., land tenure score; women's and men's scores on the five dimensions of empowerment).

## Limitations

The field survey was conducted within two major regional states of Ethiopia. Therefore, it may not reflect the situations in other regions and contexts of the country. The study was conducted in predominantly lowland agroecologies in Oromia and SNNP regional states. The findings generated from this study may not be easily applied to other agroecologies, such as the midlands and highlands in Ethiopia. The data was cross-sectional, collected towards the end of the rainy season and, hence, it may not reflect the situation throughout the year and provides limited insight into household dynamics and other changes in the target territories over a longer period.

## Ethics Statement

The entire research was conducted following the highest ethical and standard operating procedures (SOPs) of safeguarding the rights, safety and wellbeing of all human subjects participating in the research. This project will pose minimal health risk on human subjects participating in the research activities since it does not involve the collection of blood, urine or other samples directly from the respondent. The research team practiced informed consent during data collection to safeguard privacy and anonymity, and ensure overall ethical acceptability/integrity of the research.

## CRediT authorship contribution statement

**Muluken G. Wordofa:** Conceptualization, Methodology, Software, Formal analysis, Visualization, Data curation, Writing – original draft, Writing – review & editing, Funding acquisition. **Chanyalew S. Aweke:** Conceptualization, Methodology, Investigation, Supervision, Data curation, Writing – original draft, Writing – review & editing, Funding acquisition. **Getachew S. Endris:** Conceptualization, Methodology, Investigation, Supervision, Data curation, Writing – original draft, Writing – review & editing, Funding acquisition. **Getachew N. Tolesa:** Conceptualization, Methodology, Investigation, Supervision, Data curation, Writing – original draft, Writing – review & editing, Funding acquisition. **Tesfaye Lemma:** Conceptualization, Methodology, Investigation, Supervision, Data curation, Writing – original draft, Writing – review & editing, Funding acquisition. **Jemal Y. Hassen:** Conceptualization, Resources, Supervision, Writing – review & editing. **Dario Lucantoni:** Conceptualization, Resources, Supervision, Writing – review & editing. **Anne Mottet:** Conceptualization, Resources, Supervision, Writing – review & editing.

## Data Availability

Economic, Environmental and Social Performance of Agroecology in Ethiopia (Original data) (Mendeley Data)

## Appendix Supplementary materials

Image, application 1
